# The impact of chronic pre-dialysis hyponatremia on clinical outcomes in maintenance hemodialysis patients

**DOI:** 10.1007/s11255-022-03241-1

**Published:** 2022-07-02

**Authors:** Zhoucang Zhang, Liren Zheng, Yujing Pan, Mei Wang

**Affiliations:** 1 Department of Nephrology, Peking University International Hospital, Beijing, 102206 China; 2 Department of Nephrology, Peking University People’s Hospital, 11 Xizhimennan Street, Xicheng District, Beijing, 100044 China

**Keywords:** Hemodialysis, Hyponatremia, Mortality, Major adverse cardiovascular events, Hyperglycemia, Fluid overload, Malnutrition

## Abstract

**Objective:**

Chronic pre-dialysis hyponatremia is not rare in maintenance hemodialysis (MHD) patients. However, the association between chronic pre-dialysis hyponatremia and mortality is uncertain due to multiple potential confounders such as hyperglycemia, fluid overload, and malnutrition. This study aimed to more comprehensively evaluate the association between chronic pre-dialysis hyponatremia and clinical outcomes in MHD patients.

**Methods:**

We analyzed the data of 194 MHD patients with regular real-time measurements of pre-dialysis serum sodium from July 2015 to March 2021. Hyponatremia was defined as SNa ≤ 135 mmol/L and normonatremia as SNa > 135 mmol/L and < 145 mmol/L. We evaluated the association of baseline pre-dialysis serum sodium (SNa) and time-averaged SNa (TASNa) levels with all-cause mortality or new major adverse cardiovascular events (MACE) in MHD patients. Furthermore, the SNa levels were glucose, serum albumin, and fluid overload adjusted. The associations between SNa levels and all-cause mortality or new MACE were analyzed using time-varying Cox regression models.

**Results:**

Among the total of 194 patients, 24 patients died and 45 new MACE occurred during a mean 35.2-month follow-up period. The baseline pre-dialysis SNa level was 137.1 ± 2.8 mmol/L (127–144 mmol/L). Kaplan–Meier survival analysis showed that there were no significant differences in all-cause mortality or new MACE between hyponatremia and normonatremia groups according to baseline pre-dialysis SNa or glucose-corrected SNa (gcSNa). The mean values of both TASNa and time-averaged glucose-corrected SNa (TAgcSNa) were 136.9 ± 2.4 mmol/L and 138.3 ± 2.0 mmol/L, respectively. Kaplan–Meier survival analysis showed that patients with pre-dialysis hyponatremia had higher all-cause mortality or new MACE compared with normonatremia patients whether grouped on TASNa or TAgcSNa. Cox models showed an increased risk of all‐cause mortality and new MACE in MHD patients with pre-dialysis hyponatremia based on TASNa or TAgcSNa. Even after full adjustment including time-dependent age and dialysis vintage, gender, diabetes, time-averaged weight gain (TAWG), and serum albumin, patients with pre-dialysis hyponatremia based on TASNa (HR 2.89; 95% CI 1.18–7.04; model 3) or TAgcSNa (HR 5.03; 95% CI 1.87–13.57; model 3) had approximately twofold or fourfold greater risk of all-cause mortality, respectively, compared with those with normonatremia. The risk of new MACE was also significantly elevated in patients with pre-dialysis hyponatremia based on TASNa (HR 3.86; 95% CI 2.13–7.01; model 1) or TAgcSNa (HR 2.43; 95% CI 1.14–5.15; model 1). After adjustment for time-dependent age and dialysis vintage, gender, diabetes, TAWG, and serum albumin, patients with pre-dialysis hyponatremia based on TASNa (HR 2.33; 95% CI 1.16–4.68; model 3) had a higher risk of new MACE compared with those with normonatremia.

**Conclusions:**

Pre-dialysis time-averaged hyponatremia is independently associated with increased risks of all-cause mortality or new MACE in MHD patients. The baseline SNa level is not a predictor of clinical outcomes due to its variation over time. Hyperglycemia, fluid overload, and malnutrition do not have a significant impact on the risk association between chronic hyponatremia and all-cause mortality or new MACE in MHD patients.

## Introduction

Hyponatremia is one of the most prevalent electrolyte abnormalities in maintenance hemodialysis (MHD) patients, with a prevalence of 6–29%, much higher than the general population [1–7]. MHD patients experience unique pathophysiology involving fluctuations of fluid status, malnutrition, and imbalance of sodium ingestion and excretion, and are more susceptible to hyponatremia [6, 8]. In addition, hyperglycemia in diabetic hemodialysis patients can cause intracellular electrolyte-free water to metastases outside the cell and promote hyponatremia [9, 10]. Other than acute hyponatremia, it has been revealed that chronic hyponatremia may have several unfavorable impacts on dialysis patients. Some reports showed that hyponatremia was a predictor of cardiovascular mortality and morbidity in patients with heart failure, myocardial infarction, and stroke [11–13]. A study of 441 incident peritoneal dialysis patients found that patients with lower serum sodium (SNa) had a higher risk of new fatal or non-fatal cardiovascular events [14]. Cognitive impairment has a high prevalence in dialysis patients and may be associated with chronic hyponatremia [15]. A recent study in peritoneal dialysis patients demonstrated that hyponatremia was independently correlated to worse global cognitive function and executive function, which may partly be responsible for disequilibrium and gait abnormalities [16]. It has been shown that hyponatremia could stimulate inflammation by mucosal barrier breakdown through cellular edema and was an independent predictor of a higher risk of infection leading to hospitalization in MHD patients [6, 17].

Several previous studies have found that hyponatremia was associated with an increased death risk in MHD patients [1, 3, 5, 18]. However, the impact of low SNa on clinical outcomes is complex. Multiple confounding factors such as fluid overload, malnutrition, and hyperglycemia, can contribute to hyponatremia and is likewise independently related to mortality. Therefore, we attempted to more comprehensively evaluate the association of pre-dialysis SNa including baseline SNa and TASNa levels with clinical outcomes of all-cause mortality and new major adverse cardiovascular events (MACE) with adjustment for confounding factors in MHD patients.

## Materials and methods

### Data sources

**Fig. 1:**
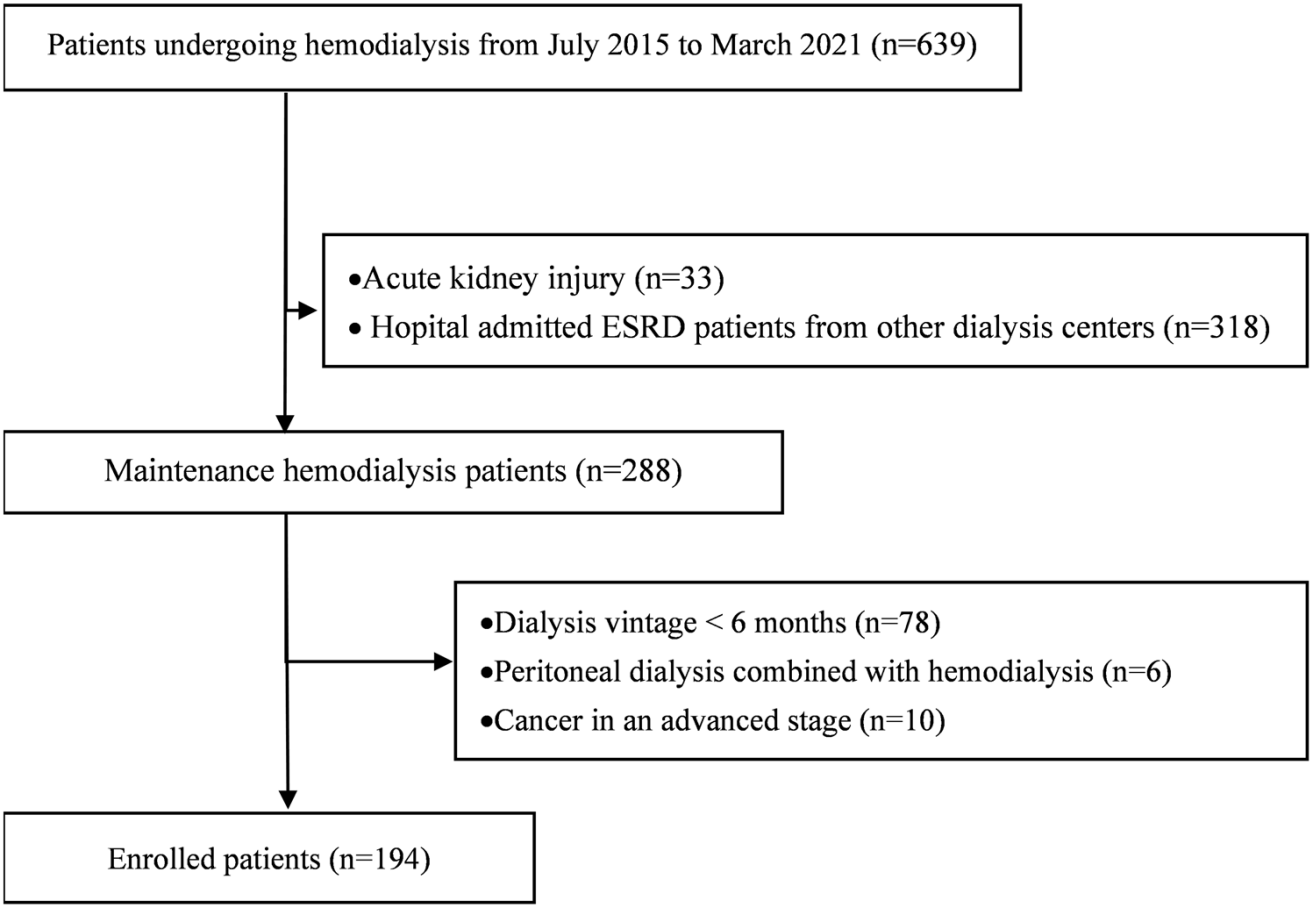
Flow diagram showing the enrollment of patients. ESRD, end-stage renal disease

We conducted a single-center retrospective cohort study. A total of 639 patients undergoing MHD in Peking University International Hospital from July 2015 to March 2021 were selected, and 194 were finally included in the study. Criteria for selecting the subjects were as follows: ① chronic renal failure with age > 18 years old; ② dialysis vintage > 6 months; ③ undergoing thrice-weekly hemodialysis across the entire study duration; ④ measurements of laboratory data carried out regularly every 1–3 months. Exclusion criteria were as follows: ① hemodialysis combined with peritoneal dialysis; ② cancer in an advanced stage. The flow diagram of patients enrollment in the study is shown in Fig. 1. A dialysate sodium prescription of 138 mmol/L was used as a conventional practice in our center. All patients were treated with high-flux dialysis. The study was approved by the Ethics Committee of Peking University International Hospital [2021-055 (BMR)]. An exemption of informed consent was obtained because of the retrospective nature of the study.

#### Laboratory data

All blood samples were taken before the start of hemodialysis sessions every 1–3 months. The time-averaged values were defined as the mean of measurements from the baseline to the end of follow-up.

#### SNa and glucose-corrected SNa (gcSNa)

SNa was measured using an indirect ion-selective electrode method by the Beckman AU5811 analyzer (Beckman Coulter Inc., USA) in our hospital center laboratory. Hyponatremia was defined as SNa ≤ 135 mmol/L and normonatremia as SNa > 135 mmol/L and < 145 mmol/L. When measured serum glucose was more than 5.5 mmol/ L, SNa value was corrected with the paired serum glucose value followed as the formula: gcSNa = SNa (mmol/ L) + 1.6 ✕ [18 ✕ serum glucose (mmol/ L) − 100]/100 [9].

#### Time-averaged weight gain (TAWG)

Interdialytic weight gain (IDWG) has been much used to assess fluid overload in MHD patients, but it was significantly different due to the length of dialysis interval. To more accurately reflect chronic exposure to fluid overload, the term TAWG was adopted in this study. TAWG was calculated by the area under the curve of IDWG over the follow-up period [19].

### Outcomes of patients

Comorbidities and outcomes of patients were obtained from the electronic medical record. The outcomes of patients included all-cause mortality and new MACE. MACE was defined as a composite end-point of non-fatal myocardial infarction, heart failure, non-fatal stroke, or all-cause mortality.

### Statistical analysis

Continuous variables were expressed as mean ± standard deviation or median with range (minimum and maximum), and differences between the two groups were tested with an independent sample *t* test. Categorical variables were expressed as n (%), and the Chi-square test was used for comparison between the two groups. Unadjusted analyses of the association between hyponatremia and individual covariates were estimated by logistic regression models.

Based on SNa, gcSNa, TASNa, and TAgcSNa, the patients were divided into hyponatremia and normonatremia groups, respectively. Kaplan–Meier curves were generated to analyze patients' all-cause mortality and MACE in the two groups. Univariate survival analysis was carried out using the log-rank test.

Time-varying Cox proportional hazards models using the conditional stepwise forward procedure were applied to analyze the risk association between TASNa or TAgcSNa and clinical outcomes, and 3 different models were constructed. Model 1: unadjusted; Model 2: adjusted for diabetes; Model 3: adjusted for diabetes plus time-dependent age and dialysis vintage, gender, TAWG, and serum albumin.

All statistical analyses were performed using IBM SPSS Statistics version 25. Statistical significance was considered at *P* < 0.05.

## Results

### Basic characteristics

The enrolled patients had a mean age of 59.4 ± 14.3 years, dialysis vintage of 5.6 ± 4.1 years, and follow-up time of 35.2 ± 17.2 months. A total of 194 patients completed 6,328 pre-dialysis laboratory tests. The median number of serum sodium tests available was 34 (4, 63) per patient. The baseline pre-dialysis SNa level was 137.1 ± 2.8 mmol/L (127–144 mmol/L). Among these patients, 51 (26.3%) had hyponatremia and 143 (73.7%) had normonatremia. No patients' pre-dialysis SNa levels were more than 145 mmol/L. Demographical, clinical, and baseline laboratory data in hyponatremia and normonatremia groups based on SNa or gcSNa are shown in Table 1.

**Table 1:** Characteristics in patients as grouped based on the baseline of SNa or gcSNa

	Overall	SNa			gcSNa		
		Hyponatremia	Normonatremia	*P*	Hyponatremia	Normonatremia	*P*
N (%)	194	51 (26.3%)	143 (73.7%)	*–*	30 (15.5%)	164 (84.5%)	*–*
Age (years)	59.4 ± 14.3	61.5 ± 14.2	58.6 ± 14.3	0.221	61.1 ± 15.6	59.1 ± 14.2	0.466
Male	63.9%	64.7%	63.6%	1.000	63.3%	64%	1.000
Vintage (years)	5.6 ± 4.1	6.1 ± 3.8	5.4 ± 4.2	0.280	6.7 ± 4.3	5.4 ± 4.1	0.129
AVA	92.3%	92.2%	92.3%	1.000	90%	92.7%	0.708
Follow-up (months)	35.2 ± 17.2	40 ± 18.5	33.6 ± 16.5	0.022	40.7 ± 18.9	34.3 ± 16.8	0.061
TAWG (kg)	2.1 ± 1.0	2.3 ± 1.1	2.0 ± 1.0	0.056	2.5 ± 1.1	2.0 ± 1.0	0.030
SBP (mmHg)	142.9 ± 15.9	145.5 ± 14.7	142.0 ± 16.4	0.178	142.6 ± 13.7	142.9 ± 16.4	0.919
DBP (mmHg)	80.9 ± 11.4	78.2 ± 11.0	81.9 ± 11.4	0.046	78.3 ± 9.9	81.4 ± 11.6	0.172
Comorbidities							
Diabetes	38.7%	47.1%	35.7%	0.181	30%	40.2%	0.316
CVD	17.0%	17.6%	16.8%	1.000	6.7%	18.9%	0.119
Laboratory tests							
SNa (mmol/L)	137.1 ± 2.8	133.4 ± 1.9	138.4 ± 1.8	< 0.001	–	–	–
gcSNa (mmol/L)	138.2 ± 2.8	–	–	–	133.5 ± 1.7	139.1 ± 1.9	< 0.001
GLU (mmol/L)	8.7 ± 4.0	10.4 ± 6.0	8.1 ± 2.8	0.011	7.4 ± 3.1	8.9 ± 4.1	0.048
ALB (g/L)	37.6 ± 4.6	37.4 ± 4.4	37.6 ± 4.7	0.792	37.4 ± 4.8	37.6 ± 4.6	0.854
CRP (mg/L)	9.0 ± 15.9	11.7 ± 21.9	8.1 ± 13.2	0.267	14.3 ± 25.6	8.0 ± 13.4	0.200
CRE (µmol/L)	825.8 ± 311	804.9 ± 314.9	833.2 ± 310.4	0.579	828.5 ± 360.4	825.3 ± 302.4	0.958
Urea (mmol/L)	24.0 ± 7.5	25.9 ± 9	23.2 ± 6.7	0.027	25.3 ± 9.91	23.7 ± 6.95	0.290
UA (µmol/L)	434.9 ± 105.9	454.3 ± 135.7	428.1 ± 92.5	0.207	463.6 ± 162.7	429.7 ± 91.6	0.276
CA (mmol/L)	2.17 ± 0.21	2.16 ± 0.23	2.17 ± 0.19	0.883	2.17 ± 0.25	2.17 ± 0.20	0.909
P (mmol/L)	1.74 ± 0.61	1.76 ± 0.65	1.74 ± 0.59	0.850	1.74 ± 0.72	1.74 ± 0.59	0.956
ALP (U/L)	92.4 ± 34.4	93.5 ± 30.3	91.9 ± 35.8	0.777	91.9 ± 32.0	92.4 ± 34.9	0.940
PTH (ng/ml)	304.2 ± 271.6	252.8 ± 184.1	322.6 ± 295.2	0.053	259.6 ± 178.4	312.4 ± 285.1	0.329
K (mmol/L)	4.89 ± 0.83	5.09 ± 1.01	4.81 ± 0.74	0.073	5.00 ± 0.98	4.86 ± 0.80	0.288
CL (mmol/L)	101.0 ± 4.2	97.8 ± 2.9	102.2 ± 4.0	< 0.001	96.7 ± 2.6	101.8 ± 3.9	< 0.001
GOT (U/L)	14.9 ± 11.9	15.6 ± 12.0	14.7 ± 11.8	0.658	17.3 ± 14.7	14.5 ± 11.2	0.328
CO_2_CP (mmol/L)	23.4 ± 3.7	22.5 ± 3.1	23.7 ± 3.8	0.043	23.1 ± 3.1	23.4 ± 3.8	0.624
HCY (µmol/L)	30.2 ± 25.4	32.9 ± 25.6	29.3 ± 25.3	0.384	34.4 ± 29.5	29.5 ± 24.6	0.327
TG (mmol/L)	2.10 ± 1.47	2.32 ± 1.54	2.03 ± 1.43	0.219	2.48 ± 1.76	2.03 ± 1.40	0.121
CH (mmol/L)	3.99 ± 0.87	4.1 ± 0.92	3.9 ± 0.85	0.465	4.17 ± 0.99	3.95 ± 0.85	0.205
β_2_-MG (mg/L)	27.4 ± 11.6	25.9 ± 7.7	27.9 ± 12.6	0.189	27.1 ± 8.2	27.4 ± 12.1	0.885
spKt/V	1.46 ± 0.3	1.45 ± 0.26	1.46 ± 0.31	0.795	1.4 ± 0.27	1.47 ± 0.31	0.248
HB (g/L)	99.9 ± 19.5	104.9 ± 19.9	98.1 ± 19.1	0.03	101.4 ± 22.2	99.8 ± 19.1	0.634

*AVA* arteriovenous access including arteriovenous fistula and arteriovenous graft, *TAWG* time-averaged weight gain, *SBP* systolic blood pressure, *DB*P diastolic blood pressure, *CVD* cardiovascular disease, *TASNa* time-averaged serum sodium, *TAgcSNa* time-averaged glucose-corrected serum sodium, *GLU* glucose, *ALB* albumin, *CRP* C-reactive protein, *CRE* creatinine, *UA* uric acid, *CA* calcium, *P* phosphate, *ALP* alkaline phosphatase, *PTH* parathyroid hormone, *K* potassium, *CL* chloride, *GOT* glutamic oxaloacetic transaminase, *CO*_*2*_*CP* carbon dioxide combining power, *HCY* homocysteine, *TG* triglyceride, *CH* Cholesterol, *β*_*2*_*-MG* β_2_-microglobulin, *HB* hemoglobin

Of enrolled 194 dialysis patients, complete data were available for 173 patients (89.2%) and 21 patients (10.8%) had partially missing data because they were transferred to other dialysis centers. For these patients, we performed telephone follow-ups to acquire information about survival and new MACE. The missing number of laboratory tests was 166 accounting for 2.6% of the total 6,328 laboratory tests.

### Survival analyses based on baseline SNa or gcSNa

Among194 patients, 24 patients died and 45 new MACE occurred during a mean 35.2-month follow-up period. Kaplan–Meier survival analysis showed that there were no significant differences in survival rates between hyponatremia and normonatremia groups according to baseline pre-dialysis SNa (Log-rank test, *P* = 0.107)or gcSNa (Log-rank test, *P* = 0.719). Meanwhile, there were no significant differences in new MACE between hyponatremia and normonatremia groups according to baseline SNa (Log-rank test, *P* = 0.082)or gcSNa (Log-rank test, *P* = 0.915).

### Survival analyses based on TASNa or TAgcSNa

**Fig. 2:**
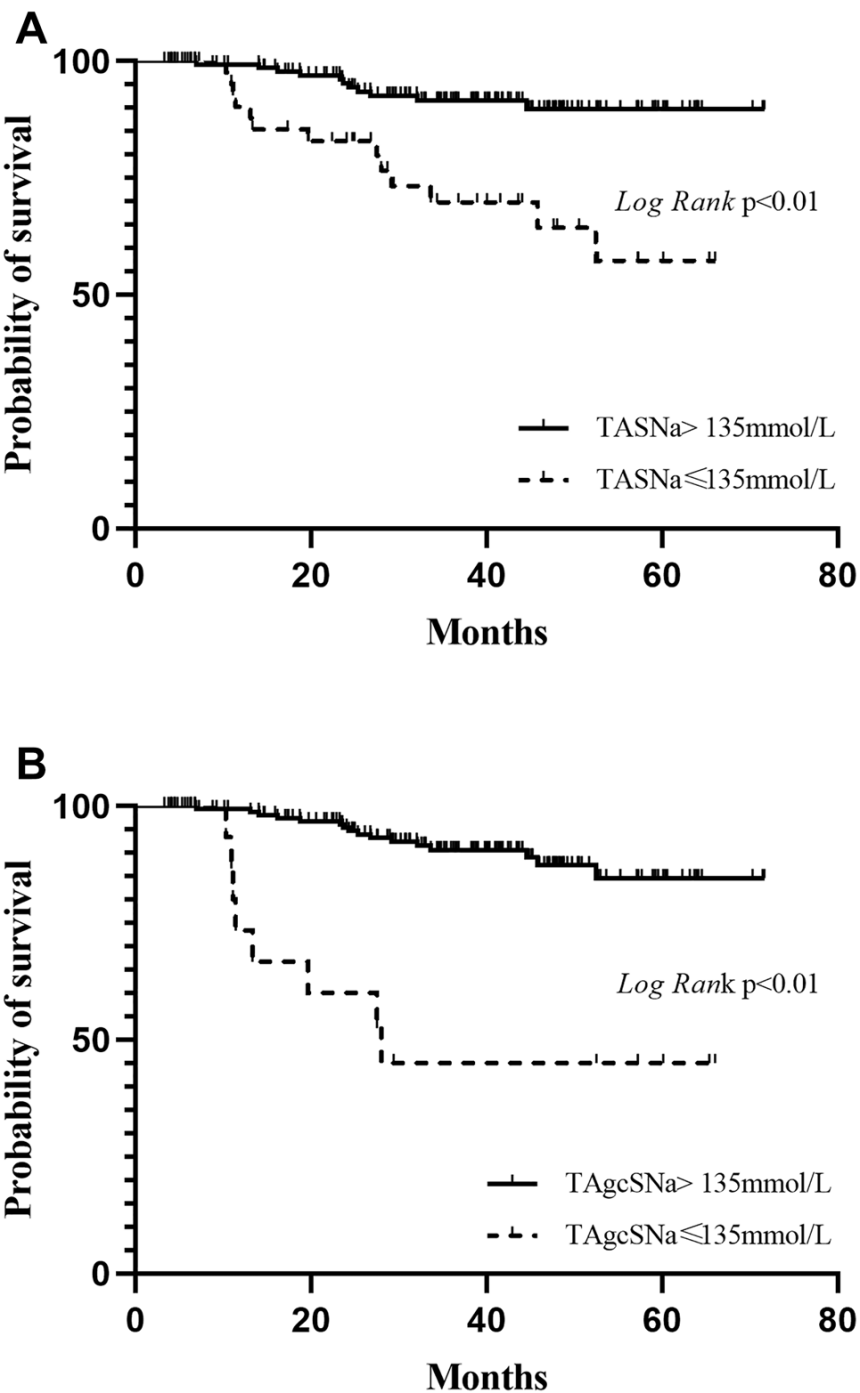
Unadjusted Kaplan–Meier curves for all-cause mortality in patients grouped on TASNa (**A**) and TAgcSNa (**B**), hyponatremia versus normonatremia; dashed lines represent hyponatremia

**Fig. 3:**
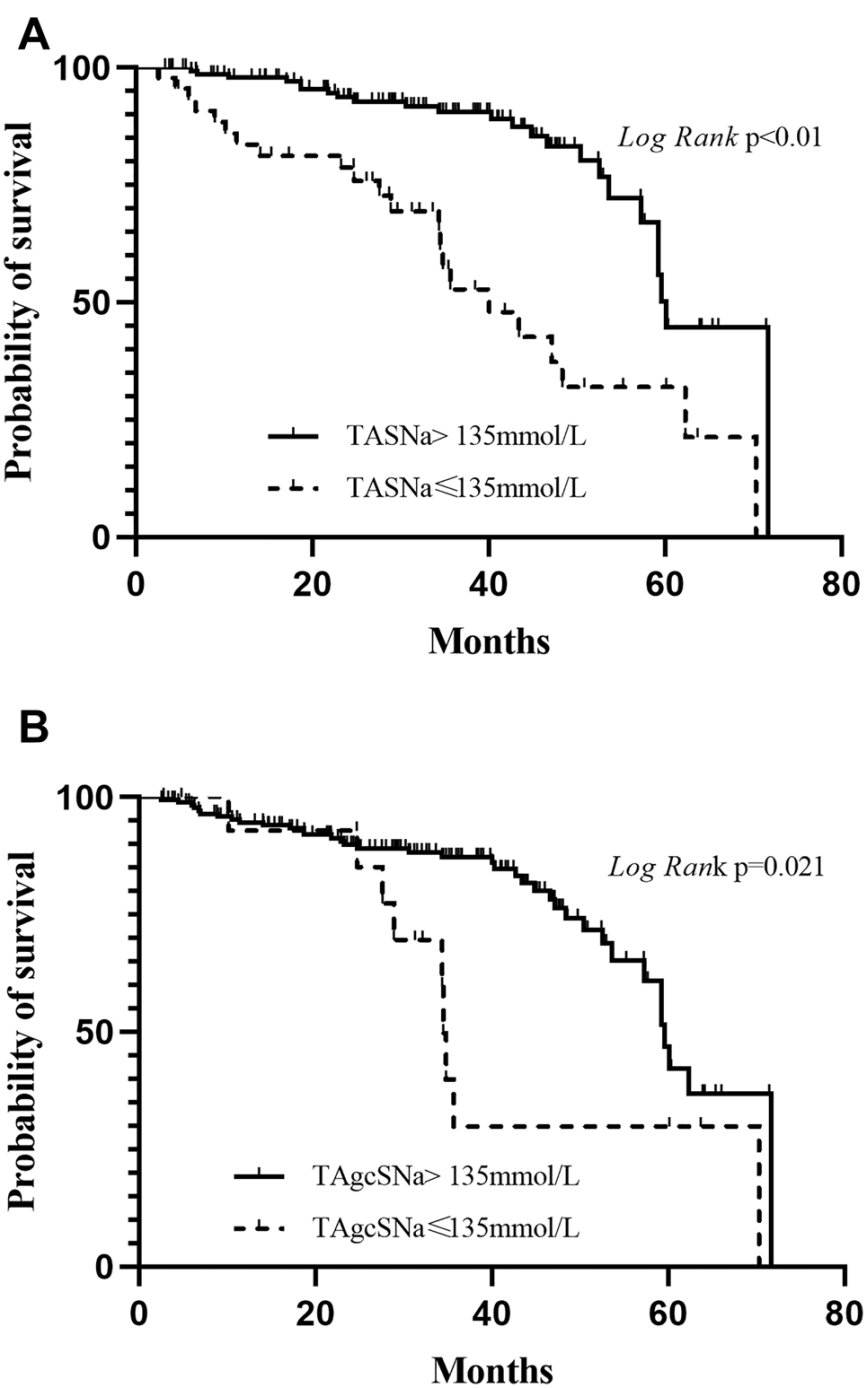
Unadjusted Kaplan–Meier curves for new MACE in patients grouped on TASNa (**A**) and TAgcSNa (**B**), hyponatremia versus normonatremia; dashed lines represent hyponatremia

The mean values of both TASNa and TAgcSNa were 136.9 ± 2.4 mmol/L and 138.3 ± 2.0 mmol/L, respectively. All values were less than 145 mmol/L. Demographical, clinical, and laboratory data in hyponatremia and normonatremia groups based on TASNa or TAgcSNa are shown in Table 2. A logistic regression analysis revealed that the patients with hyponatremia based on TASNa more likely to be female [OR 3.25 (95% CI 1.25–8.46), *P* = 0.016], and to have higher serum glucose [OR 1.39 (95% CI 1.24–1.56), *P* < 0.001], higher SBP [OR 1.06 (95% CI 1.02–1.10), *P* = 0.001] and higher CRP [OR1.09 (95% CI 1.04–1.15), *P* < 0.001]. Unadjusted Kaplan–Meier survival analysis showed that patients with hyponatremia had higher all-cause mortality compared with patients with normonatremia whether grouped on TASNa (Fig. 2A) or TAgcSNa (Fig. 2B). Meanwhile, patients with hyponatremia had higher new MACE compared with normonatremia patients whether grouped on TASNa (Fig. 3A) or TAgcSNa (Fig. 3B).

**Table 2:** Characteristics in patients as grouped based on TASNa or TAgcSNa

	Overall	TASNa			TAgcSNa		
		Hyponatremia	Normonatremia	*P*	Hyponatremia	Normonatremia	*P*
*N* (%)	194	46 (23.7%)	148 (76.3%)	*–*	16 (82.5%)	178 (17.5%)	*–*
Age (years)	59.4 ± 14.3	64.3 ± 12.8	57.8 ± 14.4	0.006	63.8 ± 14.4	58.9 ± 14.2	0.197
Male	63.9%	60.9%	64.9%	0.622	62.5%	64.0%	0.902
Vintage (years)	5.6 ± 4.1	5.5 ± 3.6	5.6 ± 4.2	0.921	6.9 ± 4.8	5.4 ± 4.0	0.158
AVA	92.3%	87.0%	93.9%	0.201	75.0%	93.8%	0.024
Follow-up (months)	35.2 ± 17.2	32.1 ± 18.1	36.2 ± 16.9	0.161	30.4 ± 22.0	35.7 ± 16.7	0.246
TAWG (kg)	2.4 ± 0.9	2.6 ± 1.2	2.3 ± 0.9	0.081	3.2 ± 1.8	2.3 ± 0.8	0.001
SBP (mmHg)	140.5 ± 15.0	147.7 ± 12.3	138.2 ± 15.1	< 0.001	147.2 ± 14.8	139.9 ± 14.9	0.062
DBP (mmHg)	78.1 ± 10.8	77.0 ± 10.2	78.4 ± 11.0	0.429	75.6 ± 10.7	78.3 ± 10.8	0.352
Comorbidities							
Diabetes	38.7%	78.3%	26.4%	< 0.001	56.3%	37.1%	0.180
CVD	17.0%	32.6%	12.2%	0.002	31.3%	15.7%	0.156
Laboratory tests							
TASNa (mmol/L)	136.9 ± 2.4	133.5 ± 1.4	137.9 ± 1.5	< 0.001	–	–	–
TAgcSNa (mmol/L)	138.3 ± 2.0	–	–	–	133.7 ± 0.9	138.6 ± 1.5	< 0.001
GLU (mmol/L)	9.4 ± 4.2	13.2 ± 4.9	8.2 ± 3.1	< 0.001	8.8 ± 2.3	9.4 ± 4.3	0.37
ALB (g/L)	38.8 ± 2.9	37.7 ± 2.8	39.1 ± 2.9	< 0.001	37.3 ± 3.3	38.9 ± 2.9	0.046
CRP (mg/L)	7.6 ± 8.4	10.2 ± 11.9	6.8 ± 6.7	0.072	17.1 ± 16.1	6.7 ± 6.7	0.022
CRE (µmol/L)	869.8 ± 232.8	745.9 ± 189.2	908.3 ± 232.1	< 0.001	764.8 ± 171.1	879.2 ± 235.6	0.06
Urea (mmol/L)	25.0 ± 3.9	24.3 ± 4.1	25.2 ± 3.8	0.188	25.73 ± 3.6	24.98 ± 3.97	0.463
UA (µmol/L)	448.2 ± 75.0	424.7 ± 79.7	455.4 ± 72.3	0.015	447.3 ± 109.3	448.2 ± 71.6	0.962
CA (mmol/L)	2.16 ± 0.15	2.16 ± 0.16	2.16 ± 0.15	0.940	2.25 ± 0.16	2.15 ± 0.14	0.010
P (mmol/L)	1.75 ± 0.37	1.65 ± 0.30	1.77 ± 0.39	0.048	1.67 ± 0.39	1.75 ± 0.37	0.447
ALP (U/L)	106.1 ± 44.8	117.4 ± 68.9	102.6 ± 33.6	0.049	133.7 ± 109.6	103.6 ± 33.1	0.291
PTH (ng/ml)	313.0 ± 182.9	280.1 ± 163.2	323.4 ± 187.8	0.161	331.4 ± 223.4	311.5 ± 179.4	0.678
K (mmol/L)	4.79 ± 0.52	4.94 ± 0.65	4.76 ± 0.46	0.072	5.36 ± 0.69	4.75 ± 0.47	0.003
CL (mmol/L)	99.3 ± 2.9	96.5 ± 2.1	100.1 ± 2.6	< 0.001	95.4 ± 1.9	99.6 ± 2.8	< 0.001
GOT (U/L)	15.3 ± 7.5	15.7 ± 5.6	15.2 ± 7.9	0.751	14.9 ± 7.2	15.4 ± 7.5	0.792
CO_2_CP (mmol/L)	23.4 ± 1.7	23.1 ± 1.4	23.4 ± 1.7	0.376	23.2 ± 1.4	23.3 ± 1.7	0.756
HCY (µmol/L)	32.5 ± 15.9	32.4 ± 17.4	32.5 ± 15.4	0.967	32.0 ± 16.7	32.5 ± 15.8	0.911
TG (mmol/L)	2.29 ± 1.25	2.38 ± 1.16	2.26 ± 1.28	0.584	2.13 ± 0.99	2.31 ± 1.27	0.593
CH (mmol/L)	3.94 ± 0.75	4.02 ± 0.92	3.91 ± 0.69	0.475	4.19 ± 0.87	3.92 ± 0.74	0.16
β_2_-MG (mg/L)	30.6 ± 8.3	30.2 ± 7.8	30.7 ± 8.4	0.733	30.9 ± 6.2	30.6 ± 8.4	0.859
spKt/V	1.55 ± 0.24	1.54 ± 0.22	1.56 ± 0.24	0.67	1.52 ± 0.21	1.56 ± 0.24	0.509
HB (g/L)	111.2 ± 10.6	110.9 ± 12.1	111.2 ± 10.1	0.908	107.5 ± 13.2	111.4 ± 10.2	0.151

*AVA* arteriovenous access including arteriovenous fistula and arteriovenous graft, *TAWG* time-averaged weight gain, *SBP* systolic blood pressure, *DB*P diastolic blood pressure, *CVD* cardiovascular disease, *TASNa* time-averaged serum sodium, *TAgcSNa* time-averaged glucose-corrected serum sodium, *GLU* glucose, *ALB* albumin, *CRP* C-reactive protein, *CRE* creatinine, *UA* uric acid, *CA* calcium, *P* phosphate, *ALP* alkaline phosphatase, *PTH* parathyroid hormone, *K* potassium, *CL* chloride, *GOT* glutamic oxaloacetic transaminase, *CO*_*2*_*CP* carbon dioxide combining power, *HCY* homocysteine, *TG* triglyceride, *CH* Cholesterol, *β*_*2*_*-MG* β_2_-microglobulin, *HB* hemoglobin

Time-varying Cox proportional hazard models showed worse outcomes in patients with hyponatremia based on TASNa or TAgcSNa (Table 3). Pearson correlation between partial residuals of variables and survival time were performed, age and dialysis vintage were considered as time-dependent variables in our study. After adjustment for time-dependent age and dialysis vintage, gender, diabetes, TAWG, and serum albumin, patients with hyponatremia based on TASNa (HR 2.89; 95% CI 1.18–7.04; model 3) or TAgcSNa (HR 5.03; 95% CI 1.87–13.57; model 3) had approximately twofold or fourfold greater risk of all-cause mortality, respectively, compared with those with normonatremia. Entering serum sodium levels as continuous variables, lower serum sodium level was associated with higher all-cause mortality risk based on TASNa (HR 0.83, 95% CI 0.70–0.99) and TAgcSNa (HR 0.81, 95% CI 0.67–0.97), respectively. The risk of new MACE was also significantly elevated in patients with hyponatremia based on TASNa (HR 3.86; 95% CI 2.13–7.01; model 1) or TAgcSNa (HR 2.43; 95% CI 1.14–5.15; model 1). After adjustment for time-dependent age and dialysis vintage, gender, diabetes, TAWG, and serum albumin, patients with hyponatremia based on TASNa had a higher risk of new MACE (HR 2.33; 95% CI 1.16–4.68; model 3) compared to those with normonatremia.

**Table 3:** Clinical outcomes risk associated with pre-dialysis TASNa and TAgcSNa

	Unadjusted			Adjusted					
	Model 1			Model 2			Model 3		
	HR	95% CI	*P*	HR	95% CI	*P*	HR	95% CI	*P*
All-cause mortality									
TASNa									
Continuous model: per 1 mmol/L higher TASNa	0.75	0.65–0.88	< 0.001	0.72	0.60–0.86	< 0.001	0.83	0.70–0.99	0.044
Categorical model									
≤ 135 mmol/L	4.32	1.94–9.65	< 0.001	5.48	2.07–14.51	0.001	2.89	1.18–7.04	0.019
> 135 mmol/L	1.00 (Reference)			1.00 (Reference)			1.00 (Reference)		
TAgcSNa									
Continuous model: per 1 mmol/L higher TAgcSNa	0.73	0.62–0.86	< 0.001	0.74	0.62–0.87	< 0.001	0.81	0.67–0.97	0.021
Categorical model:									
≤ 135 mmol/L	7.03	2.99–16.54	< 0.001	6.73	2.78–16.30	< 0.001	5.03	1.87–13.57	0.001
> 135 mmol/L	1.00 (Reference)			1.00 (Reference)			1.00 (Reference)		
MACE									
TASNa									
Continuous model: per 1 mmol/L higher TASNa	0.78	0.70–0.87	< 0.001	0.86	0.76–0.98	0.027	0.87	0.75–0.99	0.044
Categorical model									
≤ 135 mmol/L	3.86	2.13–7.01	< 0.001	2.11	1.05–4.24	0.035	2.33	1.16–4.68	0.017
> 135 mmol/L	1.00 (Reference)			1.00 (Reference)			1.00 (Reference)		
TAgcSNa									
Continuous model: per 1 mmol/L higher TAgcSNa	0.88	0.77–0.99	0.042	0.93	0.83–1.06	0.284	0.91	0.80–1.03	0.144
Categorical model:									
≤ 135 mmol/L	2.43	1.14–5.15	0.021	1.36	0.61–3.03	0.458	1.95	0.83–4.61	0.126
> 135 mmol/L	1.00 (Reference)			1.00 (Reference)			1.00 (Reference)		

Model 1 unadjusted

Model 2 adjusted for diabetes

Model 3 adjusted for diabetes plus time-dependent age and dialysis vintage, gender, TAWG, and serum albumin

*TASNa* time-averaged serum sodium, *TAgcSNa* time-averaged glucose-corrected serum sodium, *MACE* major adverse cardiovascular events

**Fig. 4:**
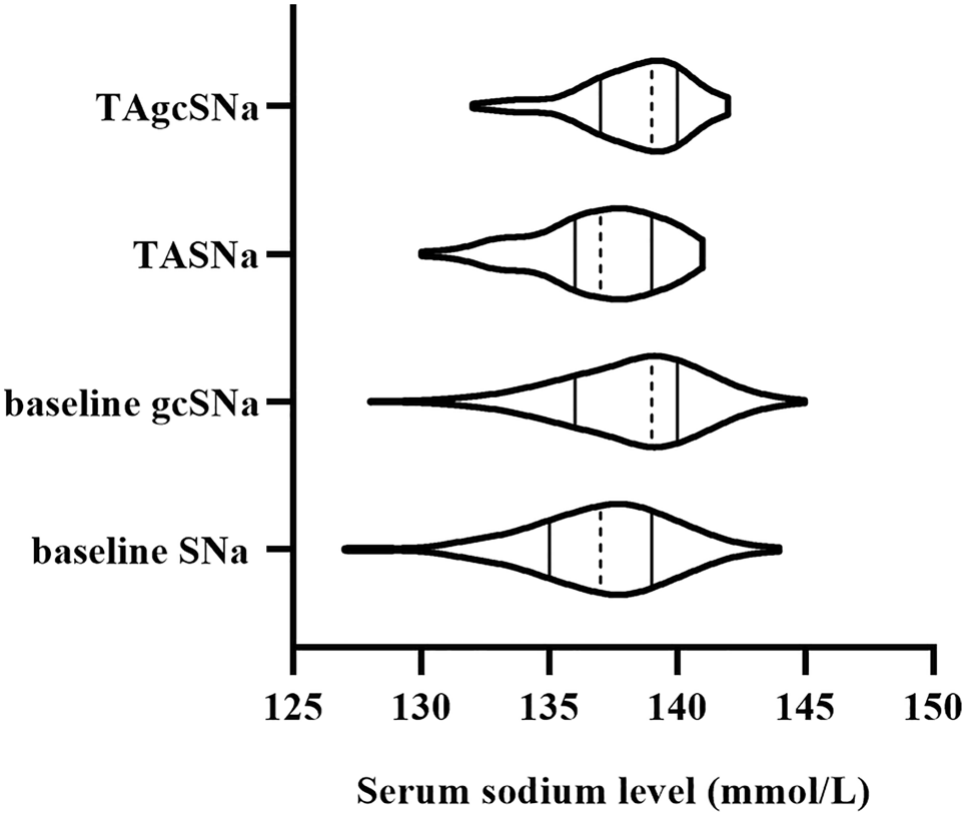
Variation of serum sodium levels during the follow-up period. The solid line represents the serum sodium quartile, and the dashed line represents the serum sodium median

Serum sodium levels including both SNa and gcSNa changed dynamically during the follow-up period. 21.6% of hyponatremia at baseline had returned to normonatremia based on TASNa, however, 6.3% of normonatremia at baseline had converted to hyponatremia based on TASNa. 50% of hyponatremia at baseline according to gcSNa had returned to normonatremia based on TAgcSNa, however, 3.7% of normonatremia at baseline according to gcSNa had converted to hyponatremia based on TAgcSNa (Fig. 4). Preliminary analysis showed that among patients with normonatremia based on baseline SNa, who converted to hyponatremia were more with diabetes (100% vs 31.3%, *P* < 0.001) and higher time-averaged glucose levels (13.0 ± 3.7 vs 8.6 ± 3.5 mmol/L, *P* < 0.001) compared with patients without conversion. Meanwhile, among patients with hyponatremia based on baseline SNa, who converted to normonatremia were less with diabetes (18.2% vs 55.0%, *P* = 0.042), lower time-averaged glucose levels (8.2 ± 3.2 vs 11.7 ± 5.4 mmol/L, *P* = 0.011), and higher time-averaged serum albumin levels (39.9 ± 1.8 vs 37.9 ± 2.9 g/L, *P* = 0.029) compared with patients without conversion.

### Subgroup analysis

We divided patients into non-diabetic (*n* = 119) and diabetic (*n* = 75) groups. Characteristics of the two groups are shown in Table 4. Cox regression revealed that higher all-cause mortality was associated with lower serum sodium levels based on TASNa in non-diabetic patients (HR 0.66, 95% CI 0.52–0.84) but not in diabetic patients (HR 0.80, 95% CI 0.63–1.02). Furthermore, TAgcSNa was used instead of TASNa in Cox regression and similar results were observed (Table 5).

**Table 4:** Characteristics in patients as grouped based on diabetes

Variables	Overall	Non-diabetic group	Diabetic group	*P*
*N* (%)	194	119 (61.3%)	75 (38.7%)	*–*
Age (years)	59.4 ± 14.3	57.4 ± 15.9	62.4 ± 10.8	0.009
Male	63.9%	56.3%	76.0%	0.006
Vintage (years)	5.6 ± 4.1	6.0 ± 4.7	4.9 ± 2.9	0.048
AVA	92.3%	91.6%	93.3%	0.786
Follow-up (months)	35.2 ± 17.2	35.2 ± 17.1	35.3 ± 17.5	0.993
TAWG (kg)	2.4 ± 0.9	2.27 ± 1.08	2.60 ± 0.73	0.013
SBP (mmHg)	140.5 ± 15	136.8 ± 15.8	146.5 ± 11.5	< 0.001
DBP (mmHg)	78.1 ± 10.8	79.1 ± 11.5	76.5 ± 9.6	0.105
Comorbidities				
CVD	17.0%	4.2%	37.3%	< 0.001
Laboratory tests				
TASNa (mmol/L)	136.9 ± 2.4	137.8 ± 2.0	135.4 ± 2.2	< 0.001
TAgcSNa (mmol/L)	138.3 ± 2.0	138.3 ± 1.9	138.2 ± 2.1	0.725
GLU (mmol/L)	9.4 ± 4.2	6.9 ± 1.3	13.3 ± 4.2	< 0.001
ALB (g/L)	38.8 ± 2.9	38.8 ± 3.2	38.7 ± 2.6	0.905
CRP (mg/L)	7.6 ± 8.4	7.5 ± 8.6	7.8 ± 8.0	0.859
CRE (µmol/L)	869.8 ± 232.8	914.5 ± 236.3	798.9 ± 209.9	0.001
Urea (mmol/L)	25.0 ± 3.9	25.6 ± 4.2	24.2 ± 3.4	0.009
UA (µmol/L)	448.2 ± 75	459.8 ± 80.4	429.6 ± 61.7	0.004
CA (mmol/L)	2.16 ± 0.15	2.19 ± 0.15	2.13 ± 0.15	0.011
P (mmol/L)	1.75 ± 0.37	1.77 ± 0.41	1.71 ± 0.31	0.280
ALP (U/L)	106.1 ± 44.8	105.7 ± 50.0	106.7 ± 35.4	0.877
PTH (ng/ml)	313 ± 182.9	331.5 ± 194.5	284.1 ± 159.5	0.078
K (mmol/L)	4.79 ± 0.52	4.72 ± 0.50	4.92 ± 0.52	0.011
CL (mmol/L)	99.3 ± 2.9	99.7 ± 2.8	98.6 ± 3.1	0.017
GOT (U/L)	15.3 ± 7.5	16.2 ± 8.8	13.9 ± 4.4	0.017
CO_2_CP (mmol/L)	23.4 ± 1.7	23.5 ± 1.7	23.1 ± 1.8	0.090
HCY (µmol/L)	32.5 ± 15.9	32.4 ± 15.0	32.6 ± 17.2	0.952
TG (mmol/L)	2.29 ± 1.25	2.21 ± 1.23	2.43 ± 1.29	0.228
CH (mmol/L)	3.94 ± 0.75	4.04 ± 0.70	3.78 ± 0.80	0.019
β_2_-MG (mg/L)	30.6 ± 8.3	32.0 ± 8.4	28.3 ± 7.6	0.002
spKt/V	1.55 ± 0.24	1.59 ± 0.24	1.48 ± 0.22	0.001
HB (g/L)	111.2 ± 10.6	110.3 ± 11.0	112.6 ± 9.7	0.140

*AVA* arteriovenous access, *TAWG* time-averaged weight gain, *SBP* systolic blood pressure, *DB*P diastolic blood pressure, *CVD* cardiovascular disease, *TASNa* time-averaged serum sodium, *TAgcSNa* time-averaged glucose-corrected serum sodium, *GLU* glucose, *ALB* albumin, *CRP* C-reactive protein, *CRE* creatinine, *UA* uric acid, *CA* calcium, *P* phosphate, *ALP* alkaline phosphatase, *PTH* parathyroid hormone, *K* potassium, *CL* chloride, *GOT* glutamic oxaloacetic transaminase, *CO*_*2*_*CP* carbon dioxide combining power, *HCY* homocysteine, *TG* triglyceride, *CH* Cholesterol, *β*_*2*_*-MG* β_2_-microglobulin, *HB* hemoglobin

**Table 5:** All-cause mortality risk associated with pre-dialysis TASNa or TAgcSNa in diabetic and non-diabetic patients

Continuous model: per 1 mmol/L higher TASNa or TAgcSNa	Non-diabetic group (*N* = 119)						Diabetic group (*N* = 75)					
	TASNa			TAgcSNa			TASNa					
	HR	95% CI	*P*	HR	95% CI	*P*	HR	95% CI	*P*	HR	95% CI	*P*
All-cause mortality	0.66	0.52–0.84	0.001	0.68	0.53–0.87	0.002	0.80	0.63–1.02	0.075	0.80	0.64–1.01	0.058

*TASNa* time-averaged serum sodium, *TAgcSNa* time-averaged glucose-corrected serum sodium

## Discussion

In this retrospective cohort study with large numbers of regular real-time measurements for laboratory values, we have investigated the association of pre-dialysis SNa with all-cause mortality or new MACE in MHD patients. Considering the effect of hyperglycemia on SNa concentration, we introduced the term gcSNa (SNa corrected with the paired serum glucose) to analyze the association between hyponatremia and survival in MHD patients. We also assessed detailed confounding factors such as fluid overload and serum albumin, which have been identified as independent predictors of mortality in dialysis patients [20–22].

The current study indicated an association between TASNa ≤ 135 mmol/L and an increased risk for all-cause mortality or new MACE in MHD patients. This finding broadly supports the work of previous studies [3–5, 18]. However, we could not observe a similar association between baseline pre-dialysis SNa and survival using Kaplan–Meier survival analysis. Otherwise, pre-dialysis SNa concentration varies over time in MHD patients. Our study revealed the mutual conversion between hyponatremia and normonatremia during the following period (Fig. 4). We speculate that this conversion may be related to diabetes, glucose, and serum albumin levels. Recent data suggested that baseline serum sodium level was not an independent predictor of 2-year mortality in peritoneal dialysis patients, partly due to the effects of several confounders including age, and nutritional status [23]. Accordingly, TASNa calculated by largely regular real-time measured SNa values may provide more substantial information about chronic hyponatremia exposure and may be more relevant to investigate the association between SNa and outcomes. Our results showed that TASNa but not baseline SNa was independently associated with increased risks of all-cause mortality or new MACE in MHD patients.

In recent years, the end-stage renal disease caused by diabetes is increasing rapidly in China [24]. Moreover, hyperglycemia may influence SNa levels by fluid shifting from the intracellular to the extracellular compartment. Cox proportional hazard models revealed that adjustment for diabetes (model 2) the risk of either all-cause mortality or new MACE was still associated with hyponatremia according to TASNa. This finding is in agreement with the study of 11,555 hemodialysis patients from the DOPPS I and III cohorts which indicated diabetes did not influence the association between lower serum sodium levels and mortality in patients with serum glucose levels < 7.8 mmol/L [5]. However, the DOPPS study was unable to demonstrate the association between hyponatremia and mortality in patients with a serum glucose level ≥ 7.8 mmol/L. In the present study, pre-dialysis SNa was corrected by paired serum glucose in patients with serum glucose > 5.5 mmol/L to understand the role of hyperglycemia on the association between hyponatremia and mortality. Our survival analysis results revealed that hyponatremia based on TAgcSNa also had an increased risk for MACE and all-cause mortality (model 1) in MHD patients. Further analyses of subgroups revealed that the risk association between serum sodium and all-cause mortality was significant in the non-diabetic population but not in diabetic patients (Table 5). Although a study of 697 hemodialysis patients indicated that low serum sodium concentration was associated with mortality only in those with diabetes, only a single serum sodium measurement might not provide accurate results to identify the patients with chronic hyponatremia [2]. As noted above, a large number of regular real-time measurements about SNa used in our study may provide more detailed longitudinal data than that of a single measurement.

Pre-dialysis SNa level may be influenced by the fluid overload that has been demonstrated to be independently associated with mortality among dialysis patients [21]. A recent large nationally representative study of incident hemodialysis patients revealed an inverse sodium-mortality association in moderate and high IDWG groups when the SNa level ranged from 136 to 139 mmol/L [18]. However, SNa levels were collected in the study over successive 91-day periods from the date of first dialysis when included patients had a high baseline comorbidity burden, which may limit the generalizability. In our study, we measured SNa before the start of hemodialysis sessions regularly every 1–3 months during the follow-up period. Using TASNa levels might reduce the effect of measured confounding variables on patients’ outcomes. Additionally, the term TAWG, which might provide a better estimate of chronic exposure to fluid overload than IDWG, was used to evaluate fluid overload in MHD patients. In the Cox proportional hazards regression, we did not find that fluid overload had a substantial effect on the relationship between hyponatremia (based on TASNa) and mortality, and new MACE in model 3.

Malnutrition has been described as a significant predictor of mortality and a potent predictor of hyponatremia in MHD patients [3, 6, 25]. This study revealed that time-averaged serum albumin and creatinine levels in the hyponatremia group were lower than in patients with normonatremia. Serum albumin and creatinine have been thought to be indices of nutrition in dialysis patients [25]. However, we did not find that serum albumin and creatinine were associated with hyponatremia in logistic regression analysis. Further Cox regression also could not reveal that serum albumin had a significant effect on the risk association between hyponatremia and all-cause mortality or new MACE in model 3.

This study has several strengths including large numbers of regular real-time measurements of pre-dialysis sodium and other variables in a single laboratory, utilization of TASNa and TAgcSNa, calculation of TAWG by the area under the curve of IDWG, and long duration of follow-up. However, several limitations need to be noted regarding the present study. First, a single-center study without hypernatremia patients and causality could not be established because of this retrospective study design. Second, we cannot exclude the possible confounding effect of residual renal function which has been recognized as a significant predictor of mortality in dialysis patients [26]. Third, there were no data on drug intake, especially diuretics. Finally, utilizing serum albumin, other than a comprehensive nutritional assessment, may not accurately evaluate the nutrition status [25, 27].

In conclusion, chronic pre-dialysis hyponatremia based on TASNa is independently associated with increased all-cause mortality or new MACE in MHD patients. The baseline SNa level is not a predictor of mortality due to its variation over time. This study demonstrates that hyperglycemia, fluid overload, and malnutrition do not have a significant impact on the association between hyponatremia with all-cause mortality, and new MACE in MHD patients. Further studies are required to increase sample size and examine the impact of residual renal function, dietary pattern, and medication, especially for diuretics. Also, nutritional evaluation by comprehensive nutritional assessment may be more appropriate in MHD patients.
